# Supplementary material for: The membrane-localized protein kinase MAP4K4/TOT3 regulates thermomorphogenesis
Source: Nat Commun. 2021 May 14;12:2842. doi: 10.1038/s41467-021-23112-0 (PMC8121802; doi:10.1038/s41467-021-23112-0)
Supplement: Supplementary file 1 — Supplementary Information [file 41467_2021_23112_MOESM1_ESM.pdf]

SUPPLEMENTARY INFORMATION

The membrane-localized protein kinase MAP4K4/TOT3 regulates thermomorphogenesis

Lam Dai Vu, Xiangyu Xu, Tingting Zhu Lixia Pan, Martijn van Zanten, Dorrit de Jong, Yaowei Wang, Tim Vanremoortele, Anna M. Locke, Brigitte van de Cotte, Nancy De Winne, Elisabeth Stes, Eugenia Russinova, Geert De Jaeger, Daniël Van Damme, Cristobal Uauy, Kris Gevaert, and Ive De Smet

Supplementary Figures

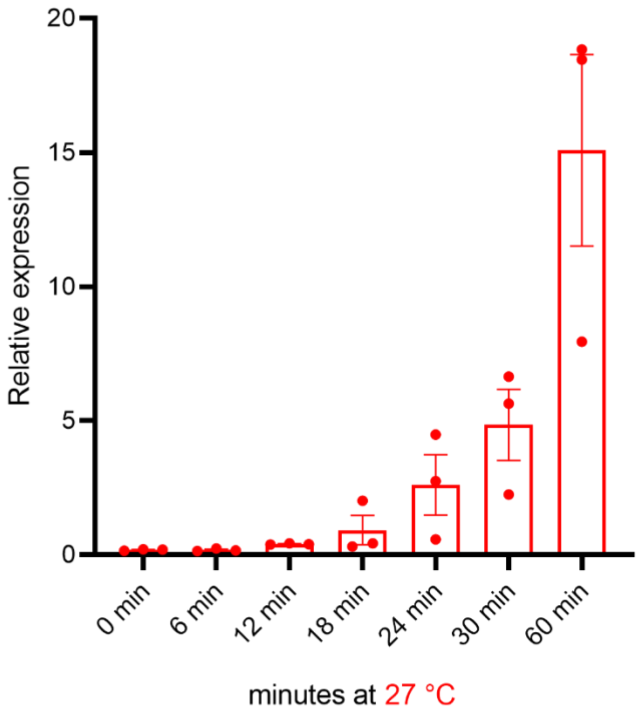

Supplementary Figure 1. Relative expression of the temperature marker *HSP70* in 10-day-old seedlings transferred from 21 °C to 27 °C for the indicated time. Bar diagram shows mean of 3 independently treated biological replicates (individual dots) with standard error of the mean.

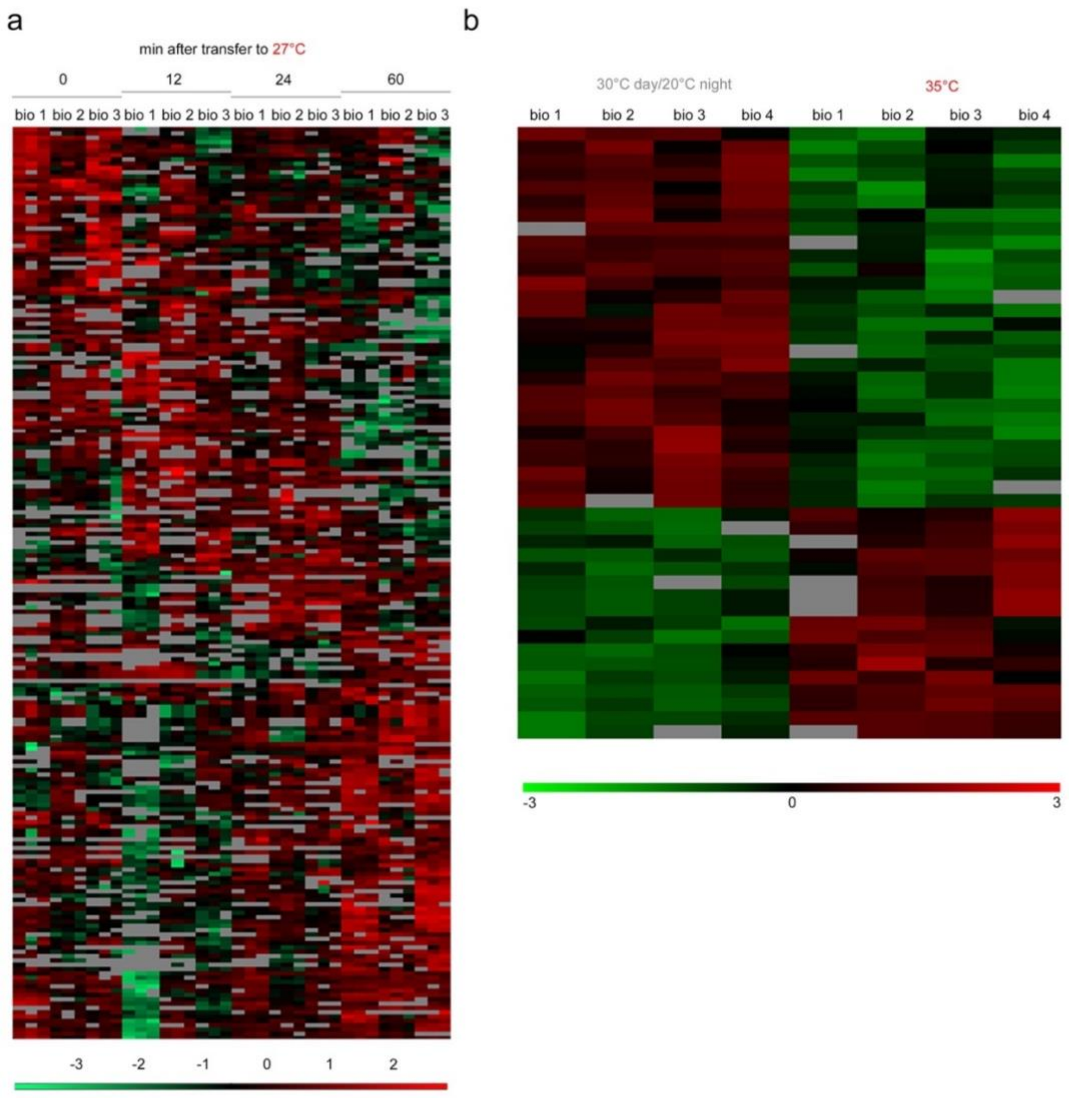

Supplementary Figure 2. Significantly regulated phosphosites in *A. thaliana* and soybean. **a,** Hierarchical clustering of 212 significantly regulated phosphosites in *A. thaliana* seedlings exposed to 27 °C. For each time point, 3 biological replicates were analyzed. For each biological replicate, 3 technical repeats were analyzed. A one-way ANOVA between the time points with a *p*-value cut-off of <0.01 was performed. **b,** Hierarchical clustering of 44 significantly regulated phosphosites in soybean leaves exposed to 35 °C compared to 30 °C control conditions. For each condition, 4 biological replicates were analyzed. Student's *t*-test with two-sample unequal variance and two-tailed distribution and a *p*-value cut-off of <0.01 was performed. Color codes indicate the phosphosite intensity from low (green) to high (red).

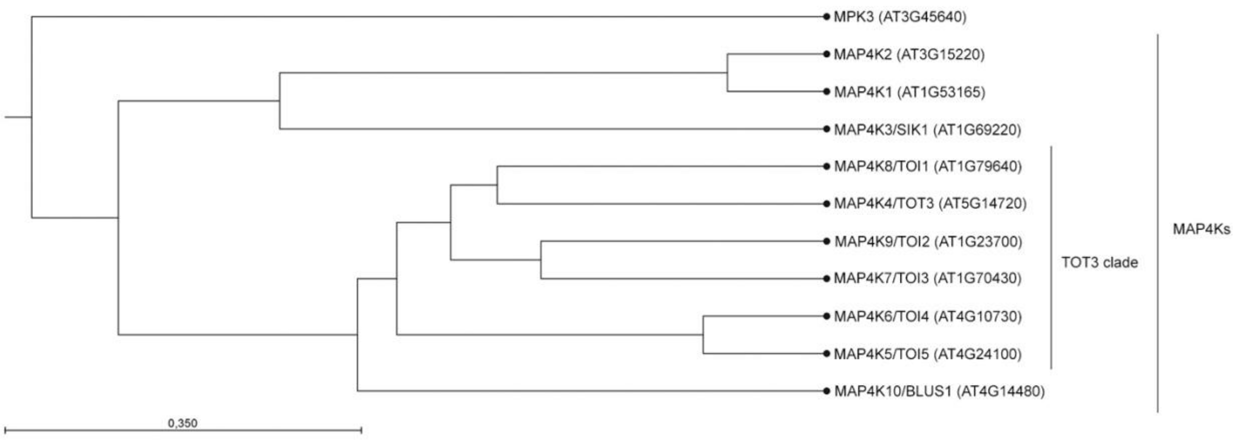

Supplementary Figure 3. Phylogram of the *Arabidopsis thaliana* MAP4K family. TOT3 clade and TOT3-INTERACTING proteins (TOIs) are indicated. The tree was reconstructed in CLC Workbench using default settings for UPGMA algorithm. Jukes-Cantor protein distance measure is indicated.

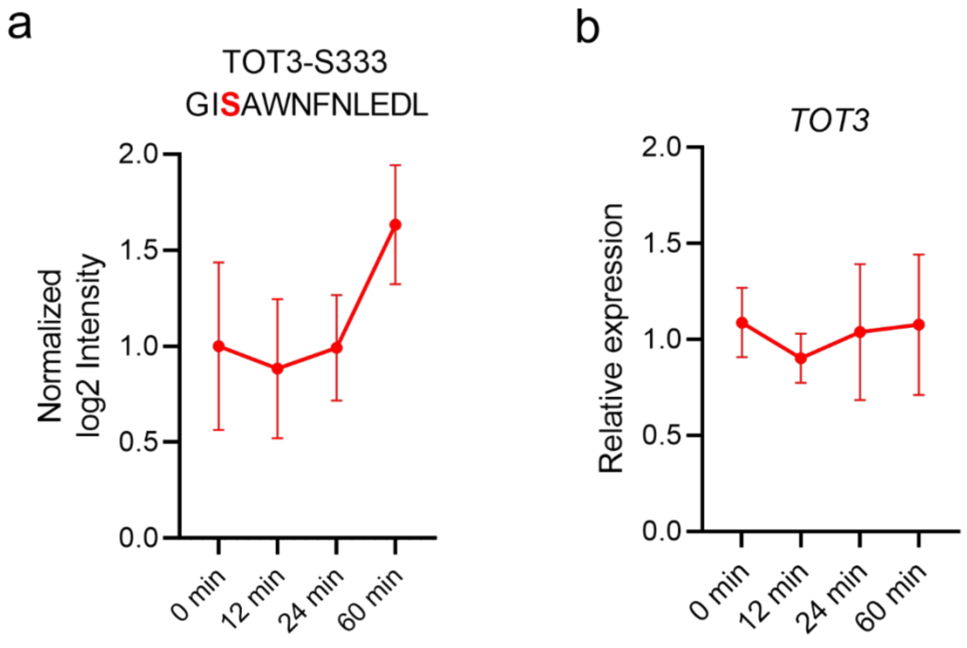

Supplementary Figure 4. a, Intensity of differentially phosphorylated TOT3 phosphopeptide containing S333 in 10-day-old seedlings upon transfer to 28 °C. **b,** *TOT3* expression in 10-day-old seedlings upon transfer to 28 °C. The mean of three biological replicates ± standard error of the mean is shown.

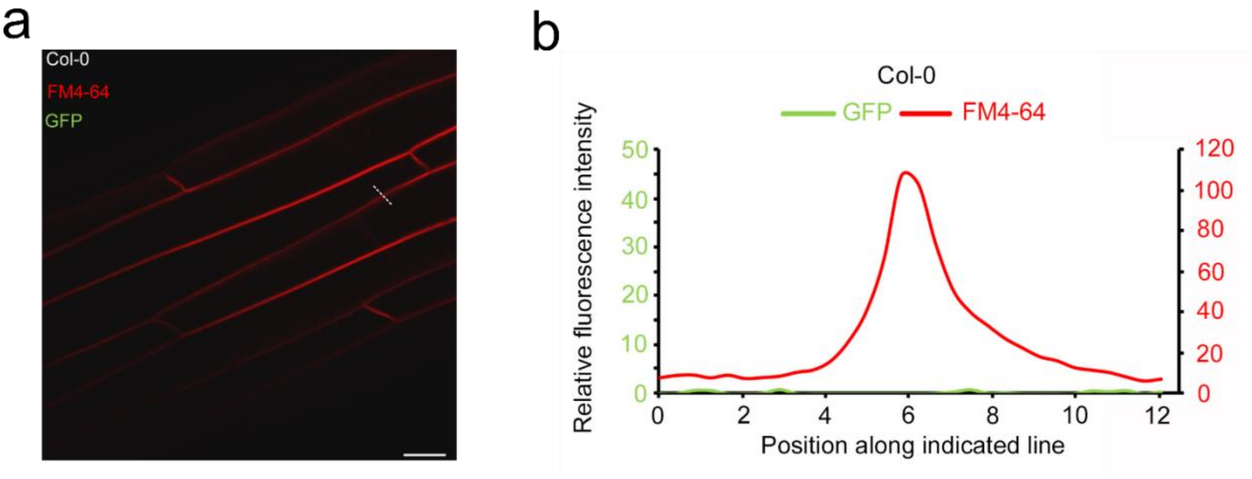

Supplementary Figure 5. a, Confocal picture of Col-0 hypocotyl stained with the endocytic tracer dye FM4-64 (red). **b,** Relative fluorescence intensity at 493-532 nm (GFP) and 596-645 (FM4-64) of Col-0 along dotted white line as the negative control for Figure 1d-e. Scale bar, 20 μm (a).

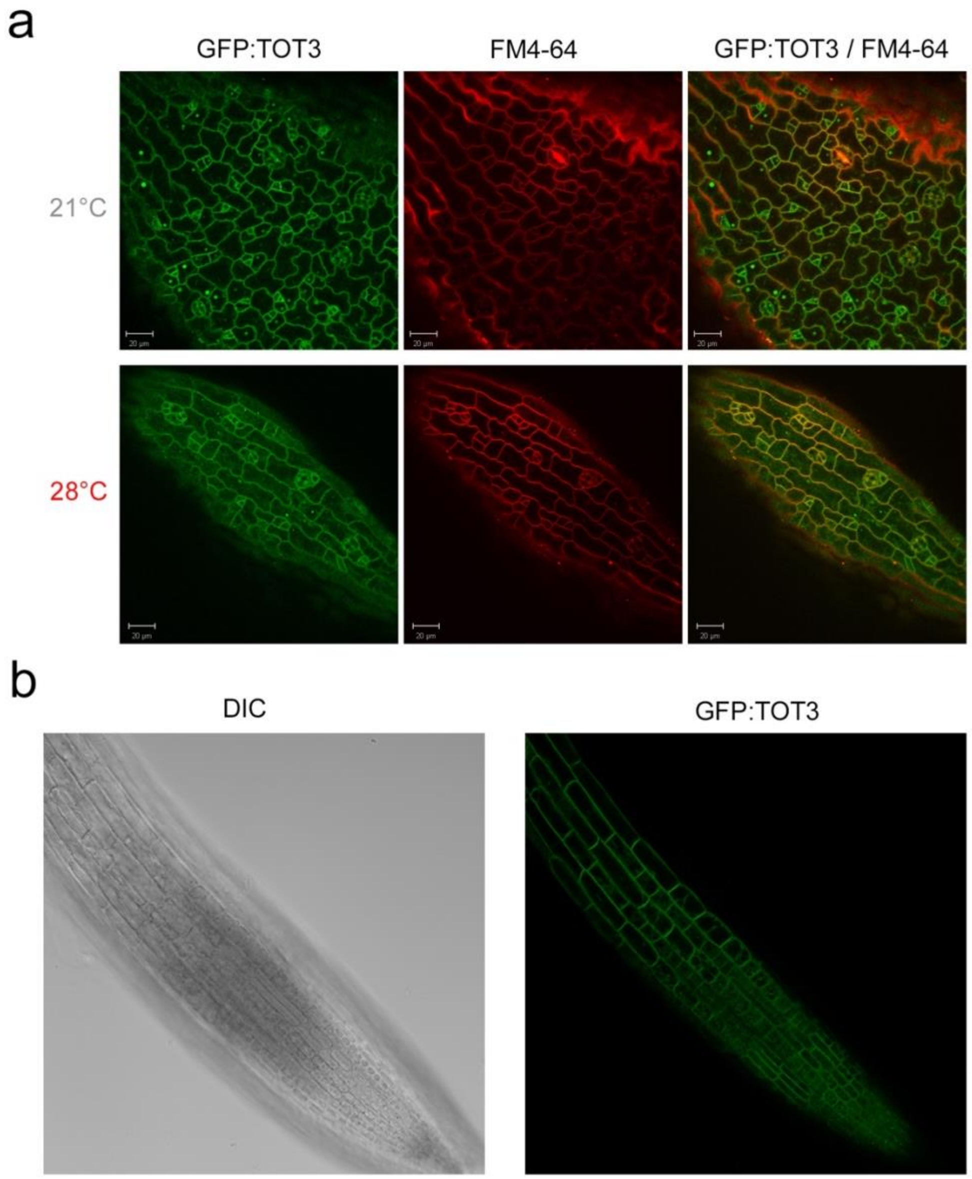

Supplementary Figure 6. TOT3 localization. **a,** Co-localization of GFP:TOT3 (green) stained with the endocytic tracer dye FM4-64 (red) in cotyledons of dark-grown *tot3-2* seedlings expressing *pTOT3::GFP:TOT3* grown at 21 or 28 °C. **b,** GFP:TOT3 (green) in root tips of *tot3-2* seedlings expressing *pTOT3::GFP:TOT3* grown at 21°C. DIC, differential interference contrast. For a-b, one micrograph is shown which is representative of at least 3 seedlings that were treated independently.

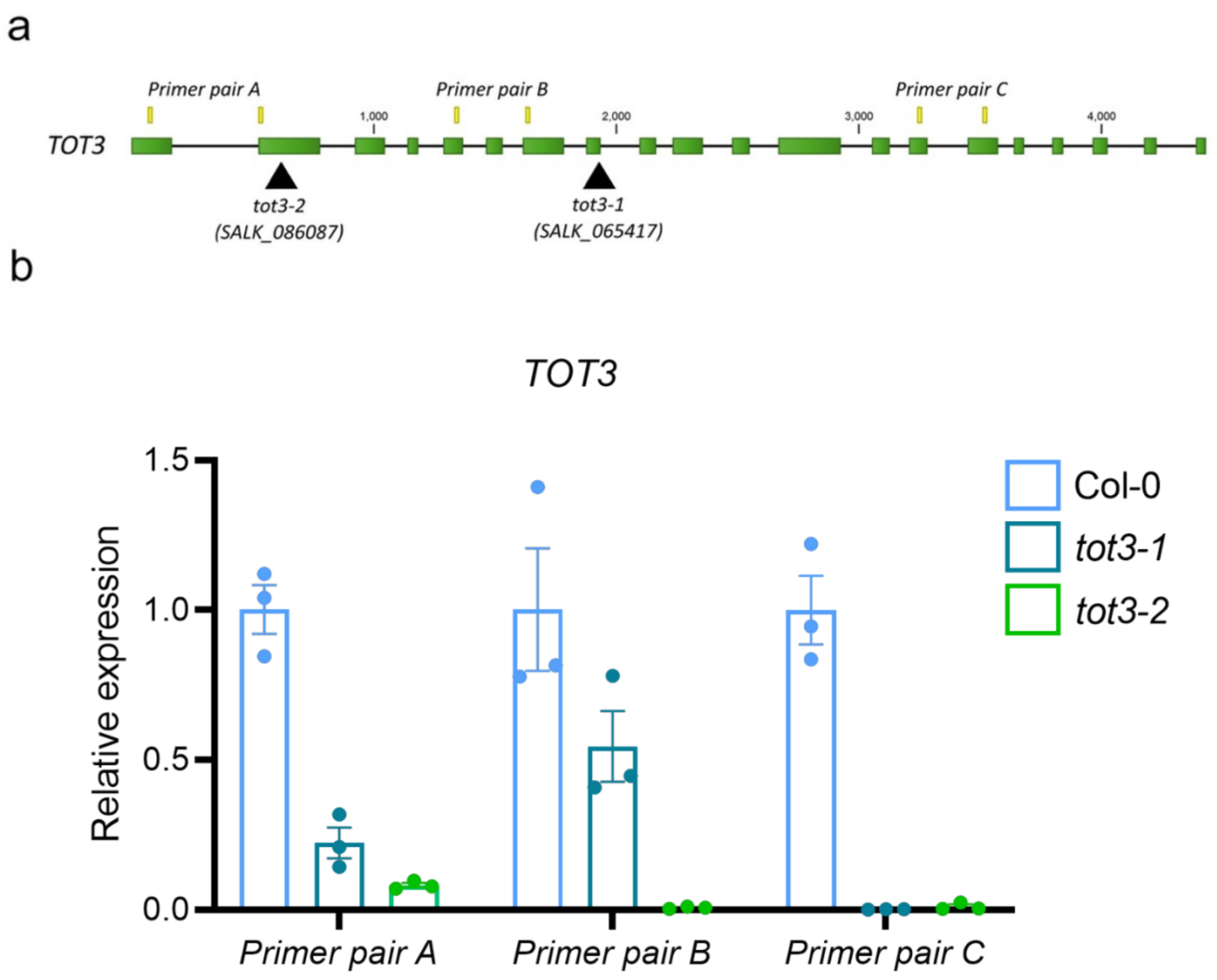

Supplementary Figure 7. *TOT3* expression in Col-0 wild type and *tot3-1* and *tot3-2* mutant lines. **a,** Position of T-DNA insertions (black triangle) and primers pairs (yellow) on the exons (green) / introns (black line) of the genomic *TOT3* sequence. **b,** *TOT3* expression using RT-qPCR primers as indicated in (a). Bar diagram shows mean of 3 biological replicates (individual dots) with standard error of the mean. Statistical analyses using Students' *t*-test with two-sample unequal variance and one-tailed distribution as indicated: *, $p < 0.05$. Average ± standard error is shown.

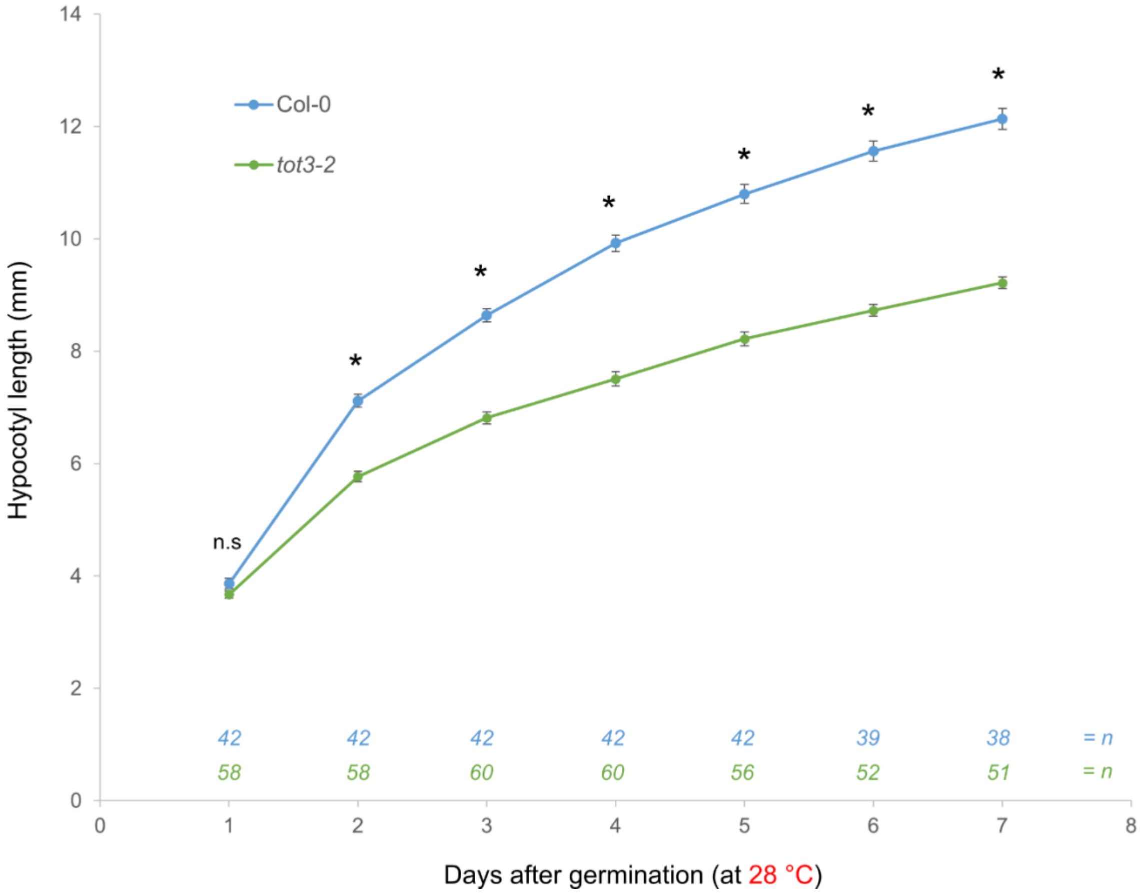

Supplementary Figure 8. Hypocotyl growth, an early hallmark of thermomorphogenesis, of Col-0 and *tot3-2*. Seven-day time course at 28 °C, following germination at 21 °C, in short-day conditions (8 h light / 16 h darkness). Statistical analyses ($n \geq 38$ seedlings) using Students' *t*-test with two-sample unequal variance and two-tailed distribution: *, *p*-value < 0.01. Mean ± standard error of the mean is shown. The number of individually measured seedlings (*n*) are indicated above the *X* axis.

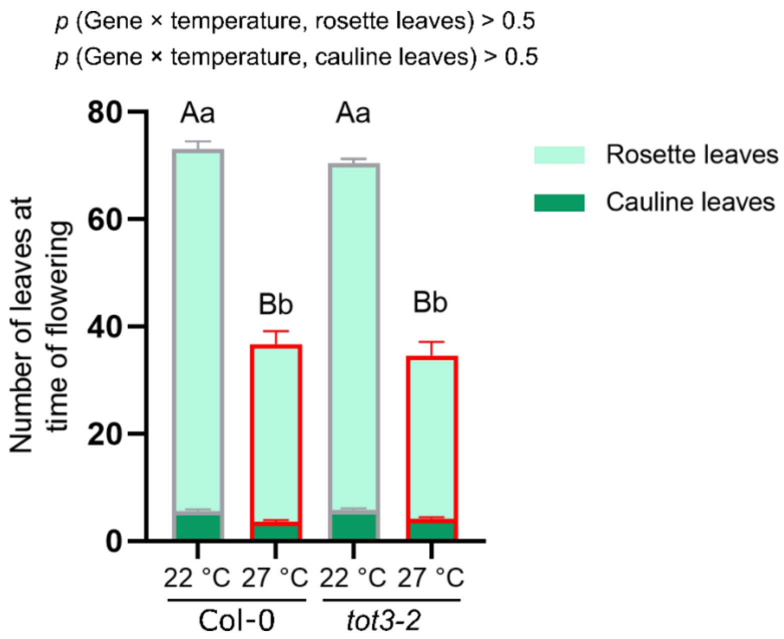

Supplementary Figure 9. Flowering time as indicated through the number of rosette and cauline leaves rosette leaves and cauline leaves at the time the first flower opens for *tot3-2* versus Col-0 at 22 °C and 27 °C. Bar charts show the mean with standard error of the mean. Leaf numbers of 32 and 27 Col-0 plants (at 22 and 27 °C, respectively) and 27 and 23 *tot3-2* seedlings (at 22 and 27 °C, respectively) were quantified. Capital and lower-case letters indicate significant differences for the rosette leaves and cauline leaves dataset, respectively, based on two-way ANOVA and Tukey's test ($p < 0.01$). The *p*-value for the interaction (genotype × temperature) is shown at the top.

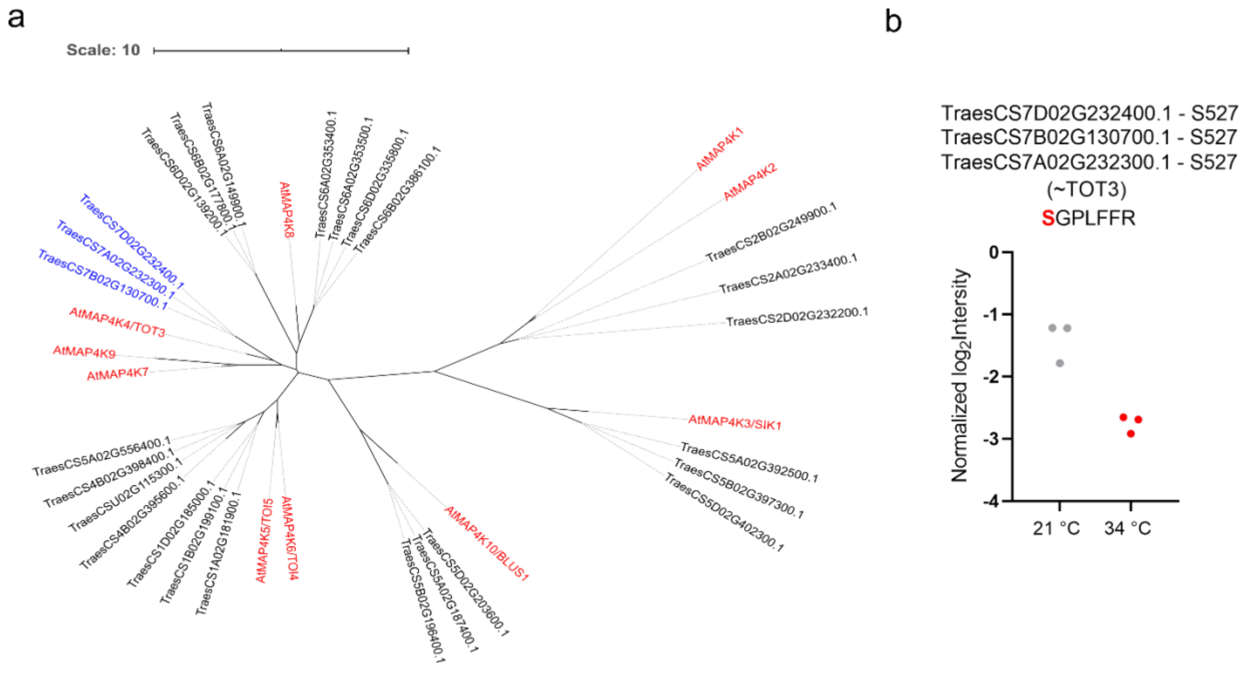

Supplementary Figure 10. a, Phylogenetic relationships between *Arabidopsis* and *T. aestivivum* MAP4Ks. *Arabidopsis* MAP4Ks are marked in red. Wheat TOT3 homologues are marked in blue. The phylogenetic tree was generated using iTOL[1]. **b,** Phosphopeptide associated with wheat TOT3 orthologs is downregulated at 34 °C compared to 21 °C in spikelets (Students' *t*-test with two-sample unequal variance and two-tailed distribution, $p < 0.01$) (data taken from ref. 2).

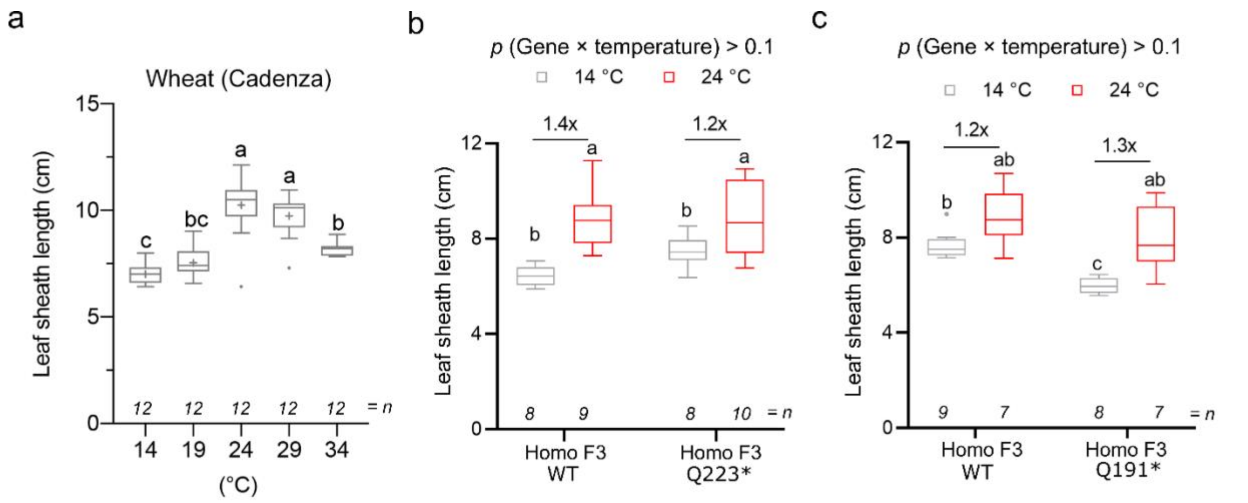

Supplementary Figure 11. a, Length of the visible leaf sheath (in those cases where the second leaf has not emerged, this leaf sheath is underneath the one of the first leaf) of two-week old Cadenza wheat seedlings grown at different temperatures, from 14 to 34 °C. **b-c,** The same measurement was performed for homozygous F3 wildtype (WT) and *tot3* two-week old Cadenza wheat seedlings. All lines were backcrossed once with wildtype Cadenza and WT plants were selected from the backcrossing with each Cadenza TILLING line (a) Q233* on TraesCS7A02G232300 (A genome) (b) Q191* on TraesCS7B02G130700 (B genome). Box plots show median with Tukey-based whiskers and outliers. Letters above the boxplots indicate significant differences based on two-way ANOVA and Tukey's test ($p < 0.01$); n.s: not significant. The p-value for the interaction (genotype × temperature) is shown at the top. The number of individually measured seedlings (n) is indicated above the X-axis.

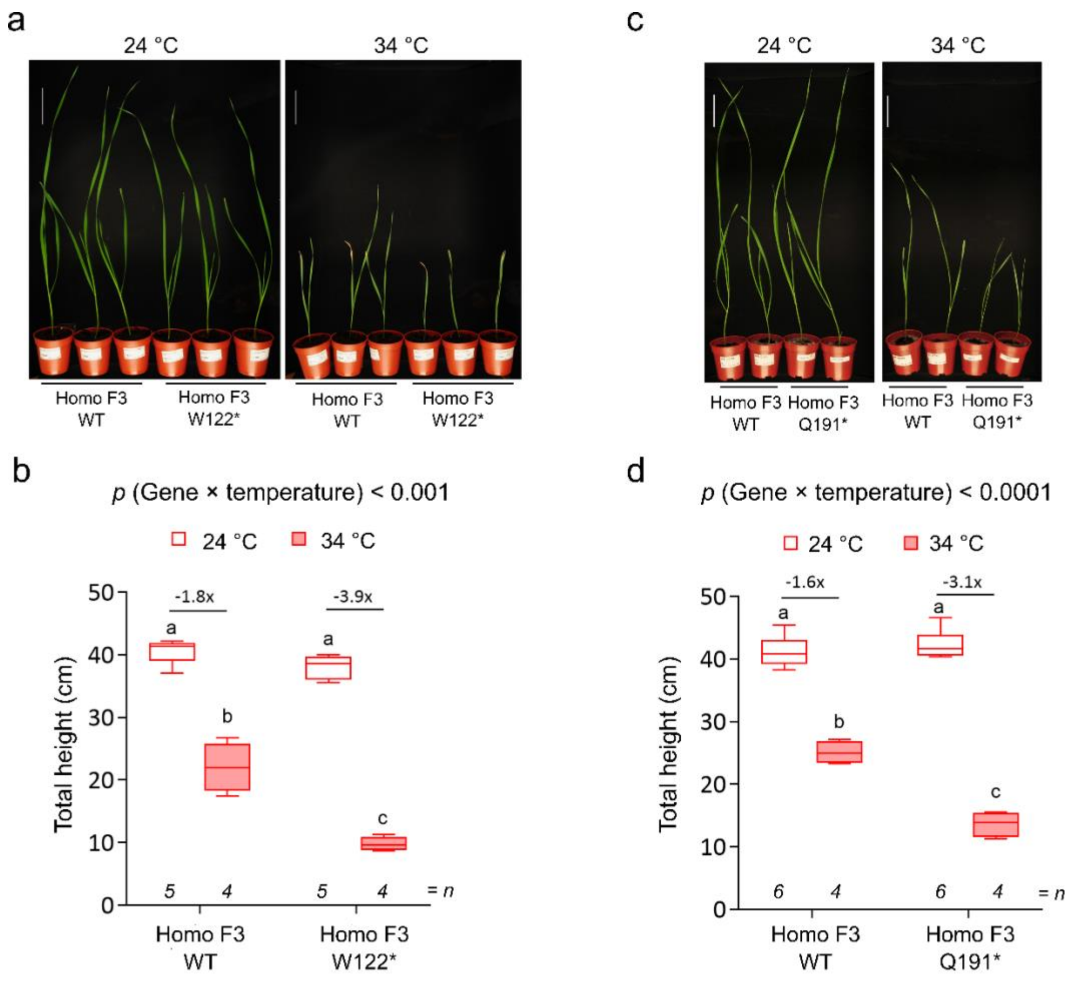

Supplementary Figure 12. Total height of W122* (a, b) and Q191* (c, d) TILLING lines from *TraesCS7D02G232400* (D genome) and their respective controls when grown at 24 °C and 34 °C for 2 weeks in long-day conditions. Representative pictures (a, c) and the respective quantification of the total height (c, d). Scale bars, 5 cm. Box plots show median with Tukey-based whiskers and outliers. Letters above the boxplots indicate significant differences based on two-way ANOVA and Tukey's test ($p < 0.01$; $n \geq 4$); n.s: not significant (c). The *p*-value for the interaction (genotype × temperature) is shown at the top. The number of individually measured seedlings (*n*) are indicated above the *X* axis.

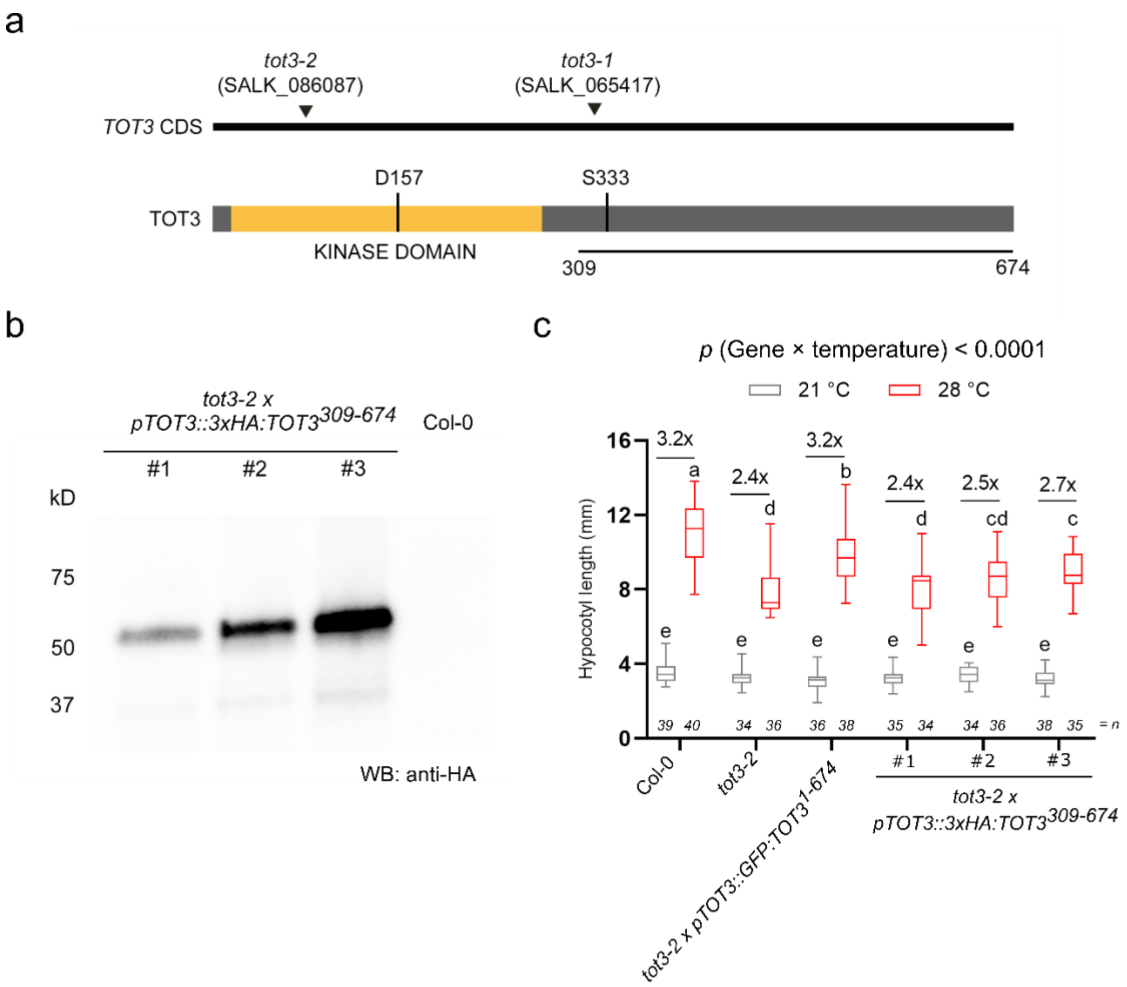

Supplementary Figure 13. TOT3 activity requires the kinase domain. **a,** Schematic representation of the *TOT3* coding sequence (CDS) and TOT3 protein sequence and domain annotation. Positions of T-DNA insertions used in this study are indicated on the CDS, with black arrowheads. The kinase domain is marked in yellow. The region for the *pTOT3::3xHA:TOT3*$^{309\text{-}674}$ construct is indicated. The positions of D157 in the conserved DFG kinase motif and S333 (significantly regulated phosphosite) are indicated. **b,** Western blot of HA:TOT3 confirming *pTOT3::3xHA:TOT3*$^{309\text{-}674}$ expression in transformed *tot3-2* seedlings. **c,** Hypocotyl length of the indicated lines at 21 and 28 °C. Box plots show median with Tukey-based whiskers and outliers. Letters above the boxplots indicate significant differences based on two-way ANOVA and Tukey's test ($p < 0.01$). The p-value for the interaction (genotype × temperature) is shown at the top. The number of individually measured seedlings (n) is indicated above the X-axis.

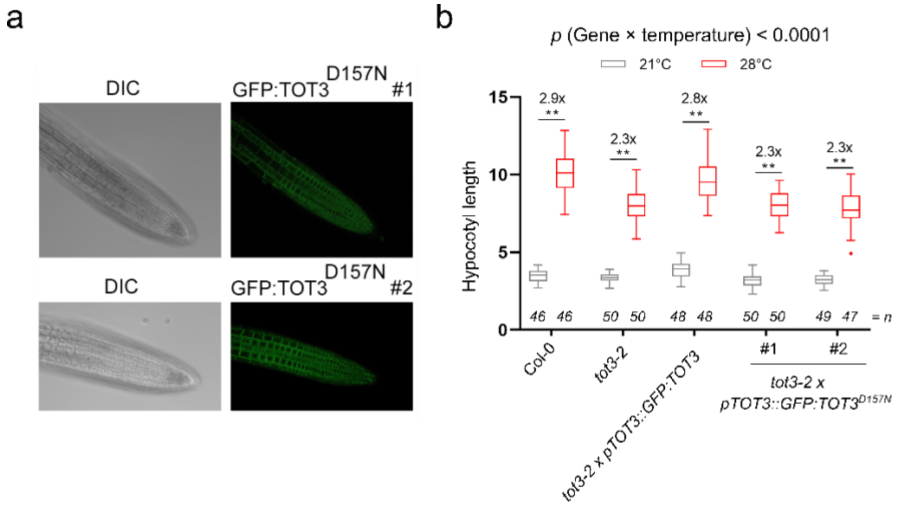

c

Phosphopeptide	Phosphosite	MBP-TOT3^{D157N}-6xHIS						MBP-TOT3^{WT}-6xHIS					
LYEEIGDGVSATVHR	S27												
LYEEIGDGVSATVHR	T29												
EADLLMQNKSEYEAHLSQQEYIR	S324												
GISAWNFNLEDLK	S333												
ASIGSLAETTKEEDTAAQNTSLPR	T466												
YLSGSIIPESTFSPKR	S488												
KYLSGSIIPESTFSPK	T489												
YLSGSIIPESTFSPKR	S491												
YQTERSYSGSLYR	S513												
SYSGSLYR	S515												
NGQLENQLNALTHR	T670												

Supplementary Figure 14. Kinase activity is required for functional TOT3. **a,** Expression of *pTOT3::GFP:TOT3*^{D157N} in *tot3-2* root tips. Representative confocal images of 3 independent seedlings for each transgenic line. **b,** Hypocotyl length of the indicated lines at 21 and 28 °C. Box plots show median with Tukey-based whiskers and outliers. Letters above the boxplots indicate significant differences based on two-way ANOVA and Tukey's test ($p < 0.01$). The *p*-value for the interaction (genotype × temperature) is shown at the top. The number of individually measured seedlings (*n*) is indicated above the *X*-axis. **c,** Phosphopeptides identified by LC-MS/MS for autophosphorylated MBP-TOT3^{WT}-6xHIS. Each column of MBP-TOT^{D157N}-6xHIS and MBP-^{TOT3WT}-6xHIS represents one replicate. The presence of phosphopeptides is marked in orange. The phosphosites are marked in red on the phosphopeptides.

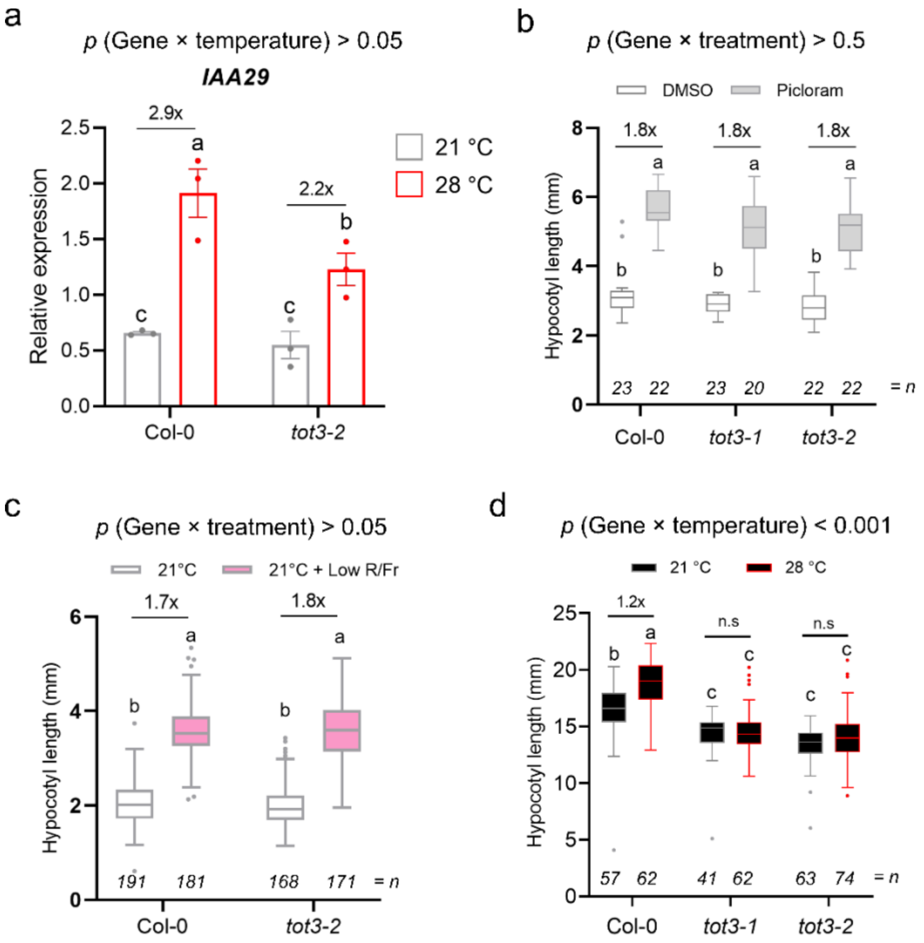

Supplementary Figure 15. a, *IAA29* expression in 3-day-old Col-0 and *tot3-2* seedlings grown at 21 and 28 °C in short day conditions. Bar charts show the mean expression of 3 biological replicates with standard error of the mean. Letters above the bar charts indicate significant differences based on two-way ANOVA and Tukey's test ($p < 0.05$). **b,** Hypocotyl length of 7-day-old Col-0 wild type and *tot3* mutants grown at 21 °C on MS/2 medium containing DMSO (control) or 5 µM picloram. **c,** Hypocotyl length of 7-day-old Col-0 wild type and *tot3* mutants grown at 21 °C in response to a low R/FR ratio light compared to control white light conditions. **d,** Hypocotyl length of 6-day-old Col-0, *tot3-1* and *tot3-2* seedlings grown at 21 and 28 °C in darkness. Box plots show median with Tukey-based whiskers and outliers. The number of individually measured seedlings (*n*) is indicated above the *X*-axis. Letters above the boxplots indicate significant differences based on two-way ANOVA and Tukey's test ($p < 0.01$). The *p*-value for the interaction (genotype × temperature or genotype × treatment) is shown at the top.

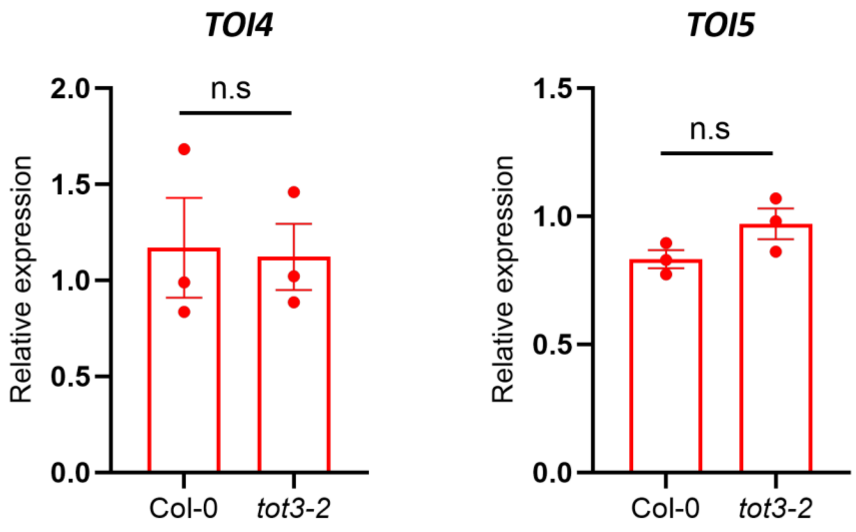

Supplementary Figure 16. Expression level of *TOI4* and *TOI5* shows no difference in Col-0 and *tot3-2* plants. Bar diagram shows mean of 3 biological replicates (individual dots) with standard error of the mean (e, f). Students't-test with two-sample unequal variance and two-tailed distribution and $p < 0.01$ was performed. n.s, not significant.

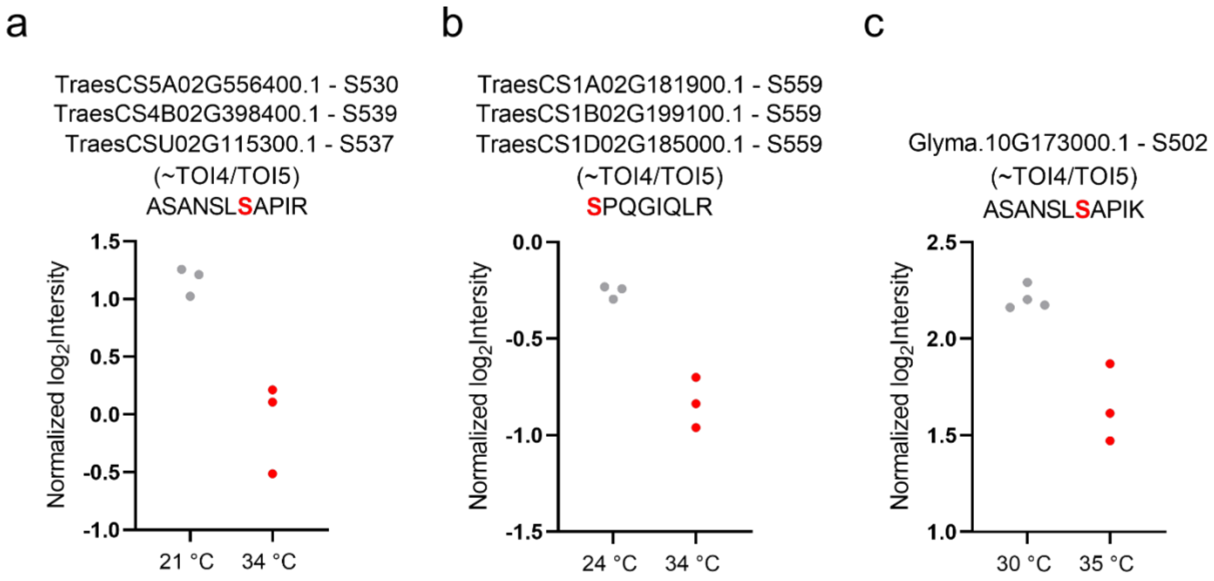

Supplementary Figure 17. Phosphosites detected for putative orthologs of TOI4 and TOI5 in wheat spikelets or seedling leaves (a-b) and soybean leaves (c), when exposed to high temperature (34 °C and 35°C for wheat and soybean, respectively), following growth at 24 °C (7 days after germination, leaf) or 21 °C (at flowering, spikelets) for wheat and 30°C (~38 days old) for soybean. Each dot represents a biological replicate. Wheat data was extracted from ref. 2. The detected phosphopeptide is indicated.

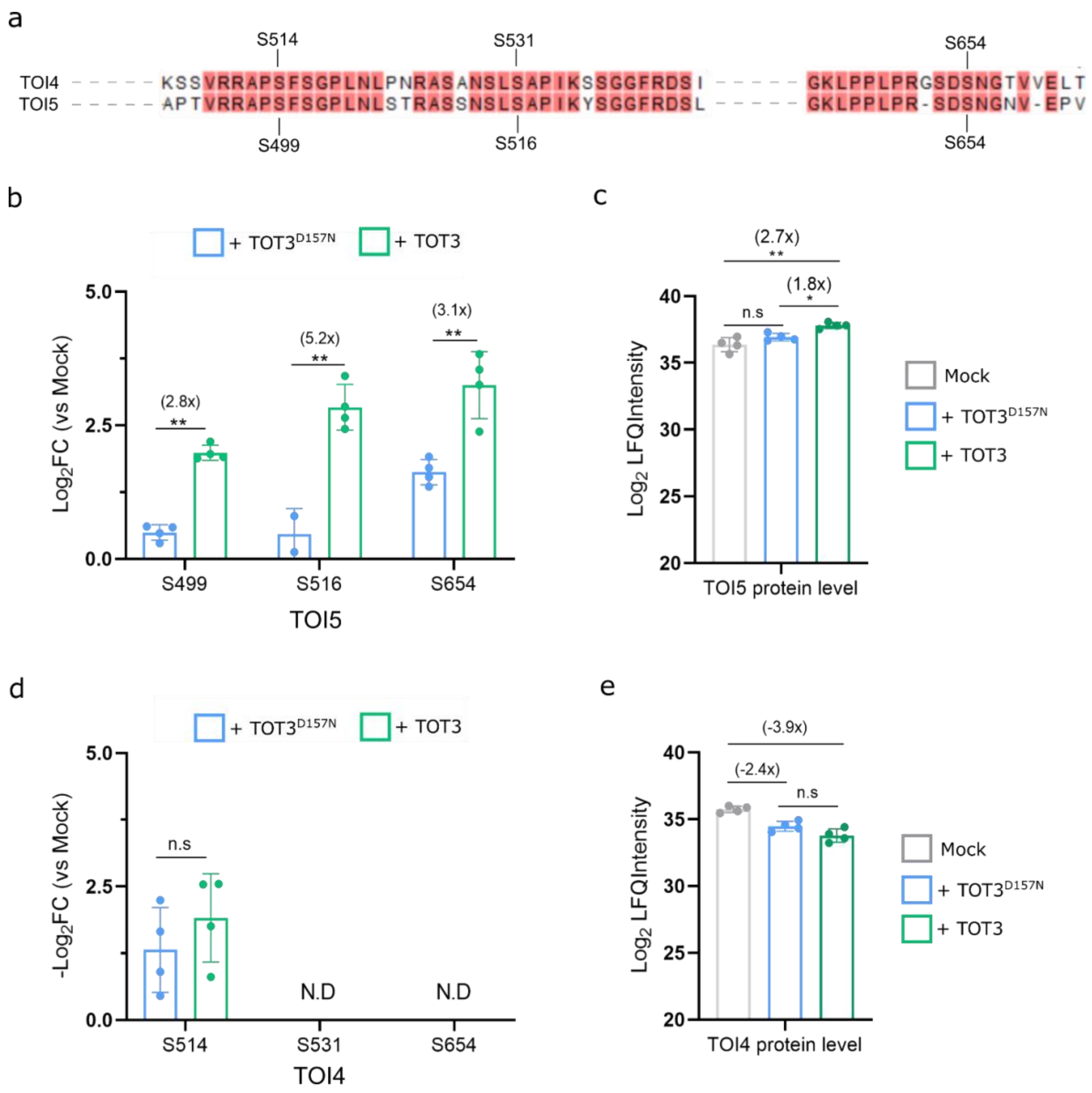

Supplementary Figure 18. Phosphorylation of RFP-TOI4^{D188N} and RFP-TOI5^{D174N} expressed in tobacco leaves. **a,** Alignment of TOI4 and TOI5 at phosphosites presented in (b) and (d). **b,** Differentially phosphorylated sites in TOI5^{D174N} co-expressed with TOT3^{D157N} or wild-type TOT3. Log2 fold change vs mock sample (without TOT3 and TOT3^{D157N}) is shown. **c,** Protein level of RFP-TOI5^{D174N} in mock and TOT3/TOT3^{D157N} co-expression samples. **d,** Differentially phosphorylated site in TOI4^{D188N} co-expressed with TOT3^{D157N} or wild-type TOT3. Two sites corresponding to those in TOI5 (see alignment in a) were not detected (N.D.). **e,** Protein level of RFP-TOI4^{D188N} in mock and TOT3/TOT3^{D157N} co-expression samples. Bar diagrams show mean of 3 biological replicates (individual dots) with standard error of the mean (b-e). Two-sample test were performed (Two-tailed Student's t-test, * $p < 0.05$, ** $p < 0.01$). n.s, not significant. The true fold change between the samples are calculated as $2^{\log_2 FC1 - \log_2 FC2}$ and indicated between the parentheses.

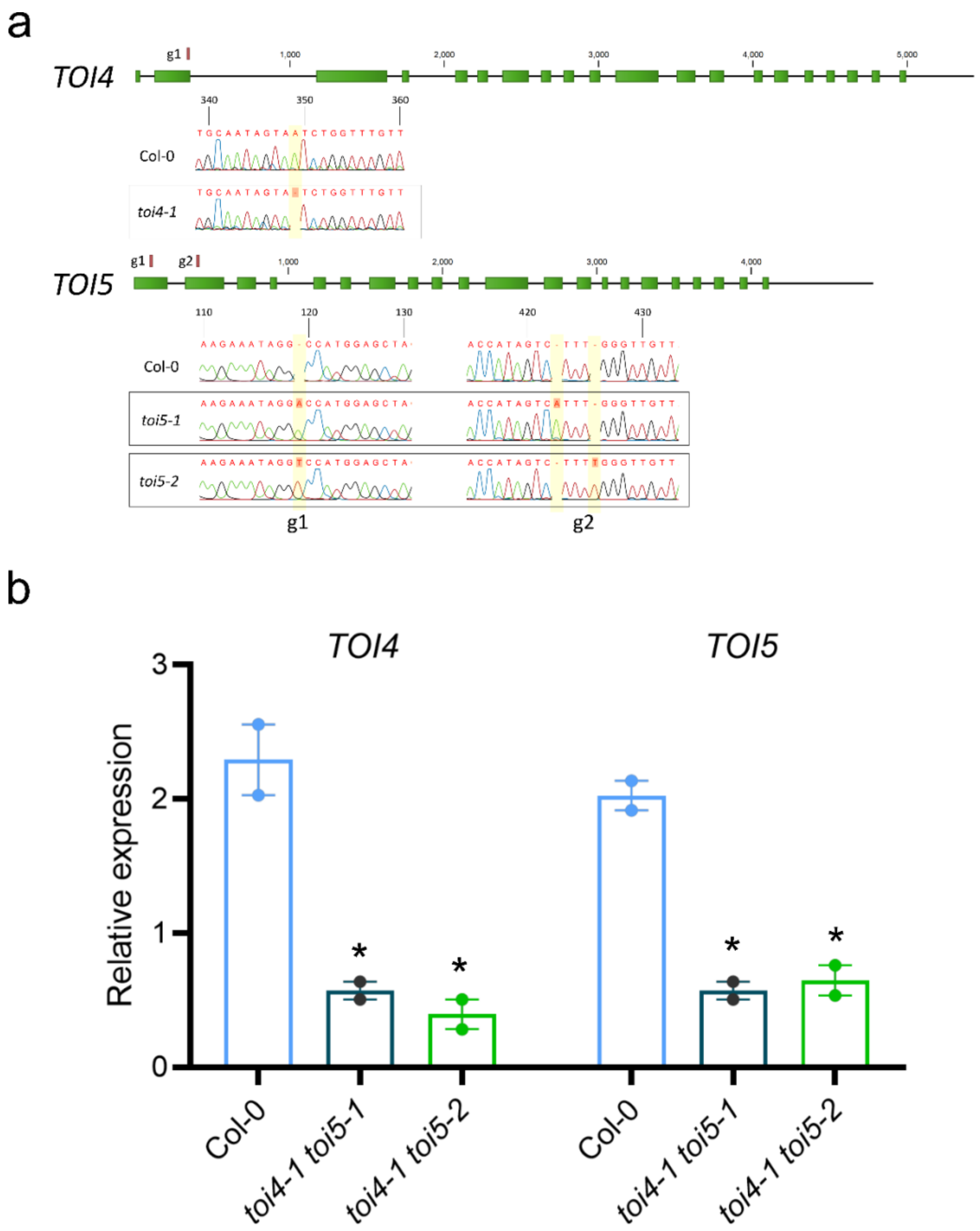

Supplementary Figure 19. a, Details of CRISP-Cas9 editing of *TOI4* and *TOI5* alleles, resulting in *toi4-1 toi5-1* and *toi4-1 toi5-2* double mutants. Exons are indicated as green bars, positions of gRNAs (g1, g2) are indicated as red line. Indel mutations are highlighted in yellow. A +1 insertion in the first and the second exons of *TOI5* caused a +2 frameshift in the CDS. A -1 deletion in the second exon of *TOI4* also caused a +2 frameshift in *TOI4* CDS. **b,** The mRNA level (as determined through qPCR) of *TOI4* and *TOI5* in *toi4 toi5* mutants. Statistical analyses using Students' *t*-test with two-sample unequal variance and one-tailed distribution as indicated: *, $p < 0.05$. Bar diagram shows mean of 2 biological replicates (individual dots) with standard error of the mean.

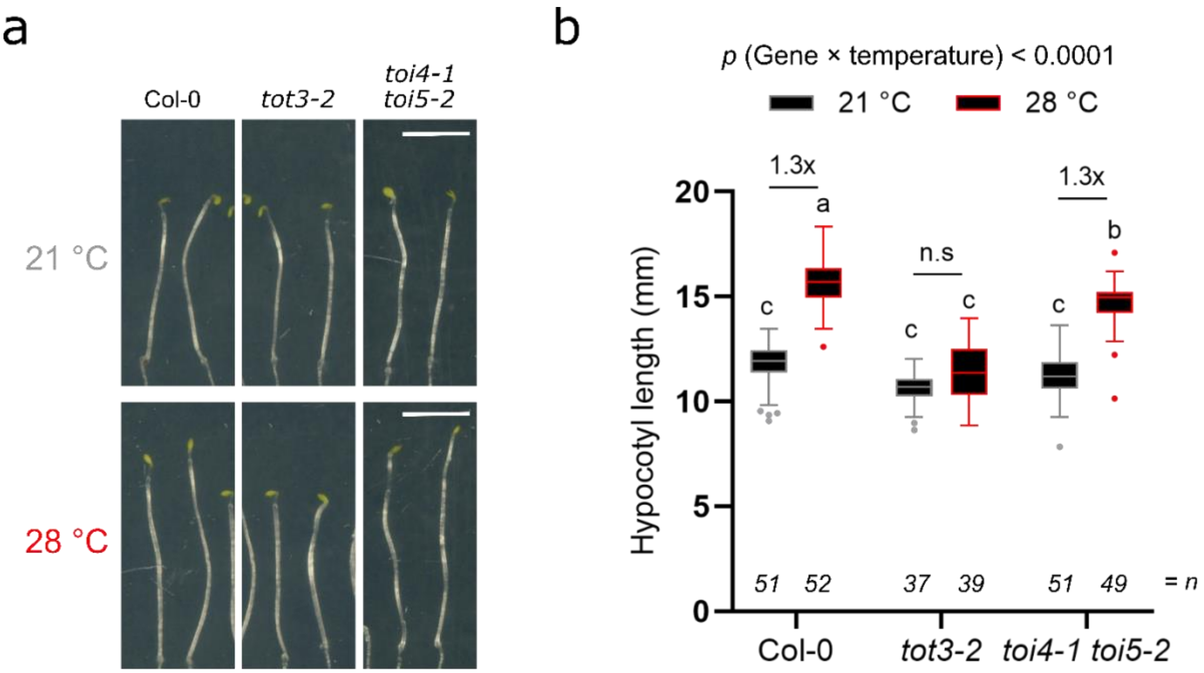

Supplementary Figure 20. Hypocotyl length of 3-day-old Col-0, *tot3-2* and *toi4-1 toi5-2* plants grown in the dark at 21 and 28 °C (a) Representative pictures. Scale bars, 5 mm. (b) Quantification of hypocotyl length. Box plots show median with Tukey-based whiskers and outliers. The number of individually measured seedlings (*n*) is indicated above the *X*-axis. Letters indicate significant differences based on two-way ANOVA and Tukey's test ($p < 0.01$) and n.s, not significant. The *p*-value for the interaction (genotype × temperature) is shown at the top**.**

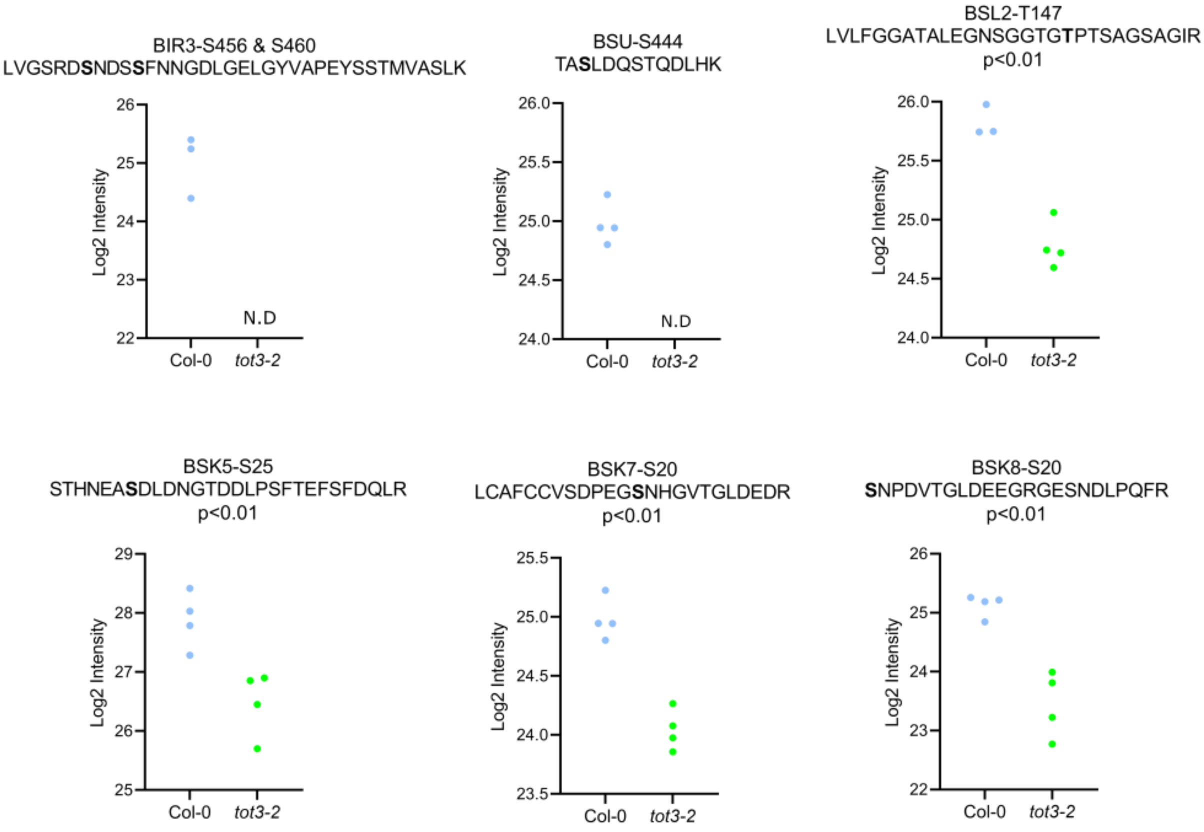

Supplementary Figure 21. Phosphosites of several brassinosteroid signalling components that are significantly deregulated in the *tot3* phosphoproteome. Each dot represents a biological replicate. Statistical analyses using Students' *t*-test with two-sample unequal variance and two-tailed distribution; *p*-value is indicated. N.D, not detected.

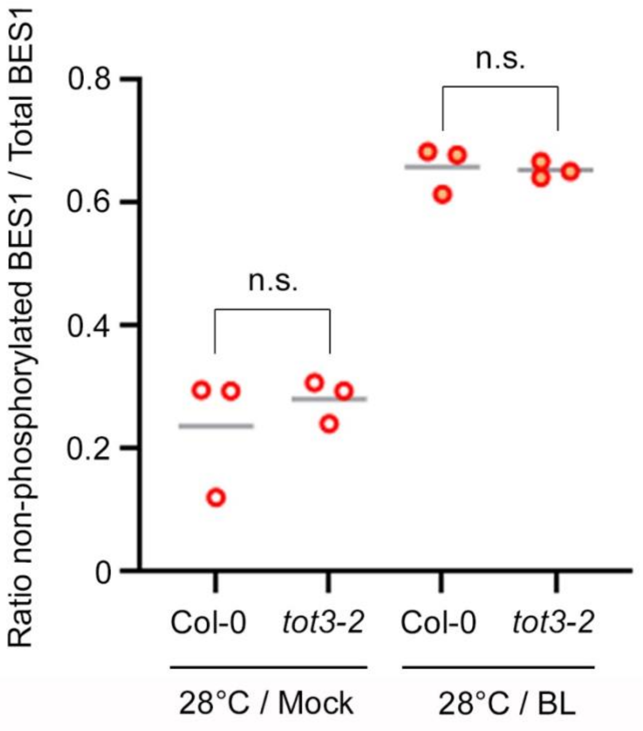

Supplementary Figure 22 Quantification of Western Blot band intensity of BES1 phosphorylation status in 4-day-old Col-0 and *tot3-2* seedlings treated with mock (DMSO) or brassinolide (BL) at 28 °C. Three independent biological replicates are shown as dots. The average is indicated through the grey line. Statistical analyses using Students' *t*-test with two-sample unequal variance and one-tailed distribution as indicated: n.s., $p > 0.5$.

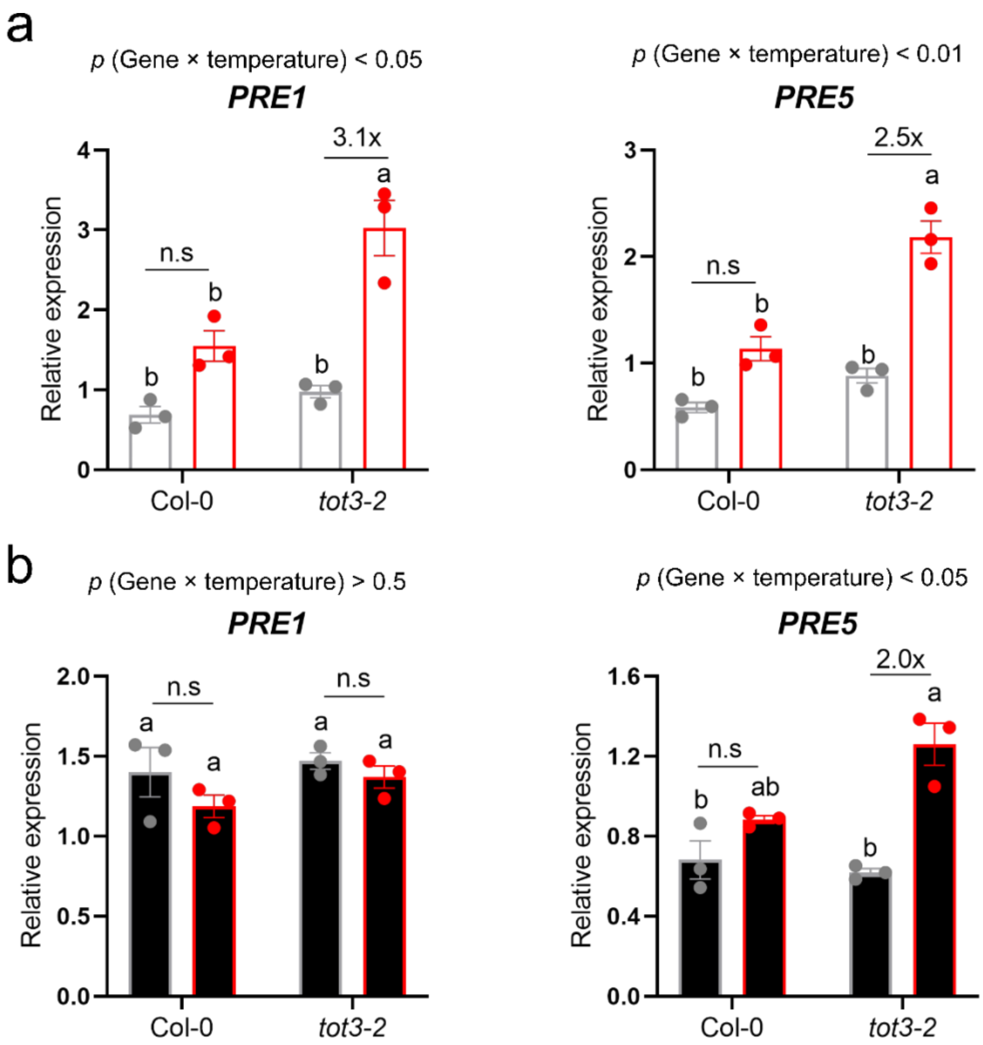

Supplementary Figure 23. Relative expression of BZR1 target genes *PRE1* and *PRE5* in 3-day-old Col-0 and *tot3-2* plants grown at control (21 °C) and warm temperature conditions (28 °C) in short day (a) and in dark conditions (b). Bar diagrams show mean of 3 biological replicates (individual dots) with standard error of the mean. Letters indicate significant differences based on two-way ANOVA and Tukey's test ($p < 0.01$) and n.s, not significant. The *p*-value for the interaction (genotype × temperature) is shown at the top.

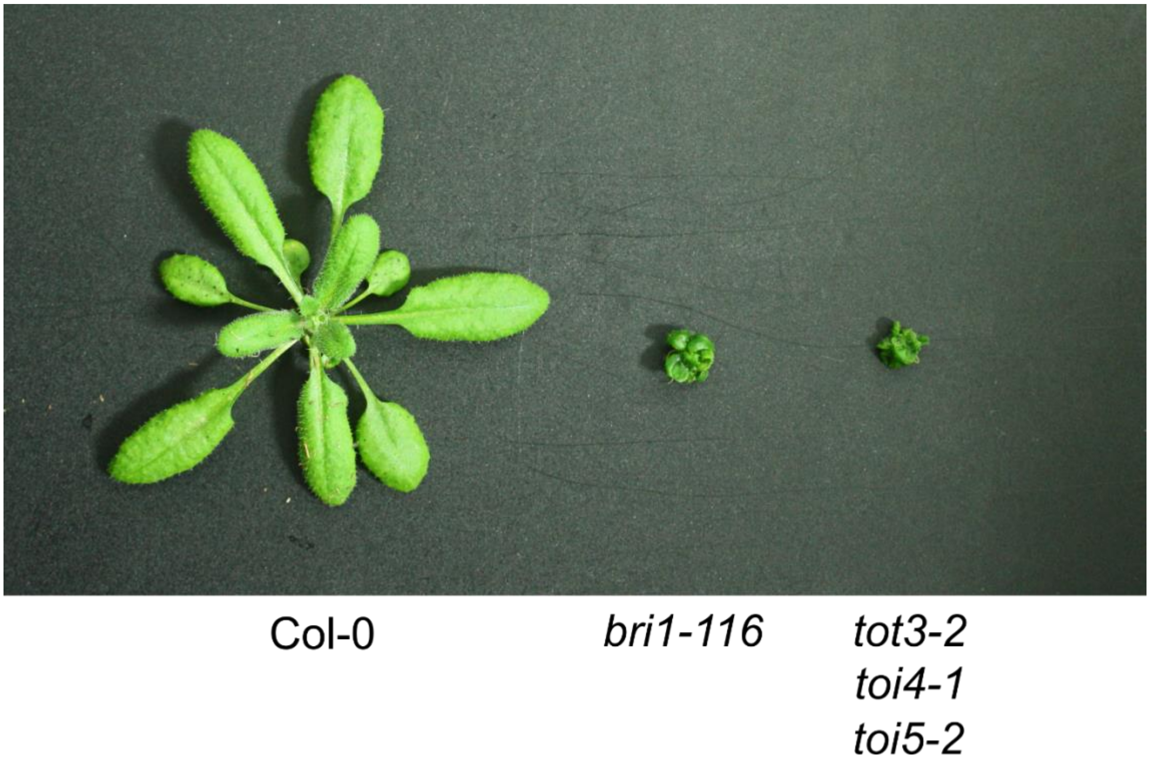

Supplementary Figure 24. 28-day-old Col-0, *bri1-116* and *tot3-2 toi4-1 toi5-1* plants grown in long day conditions.

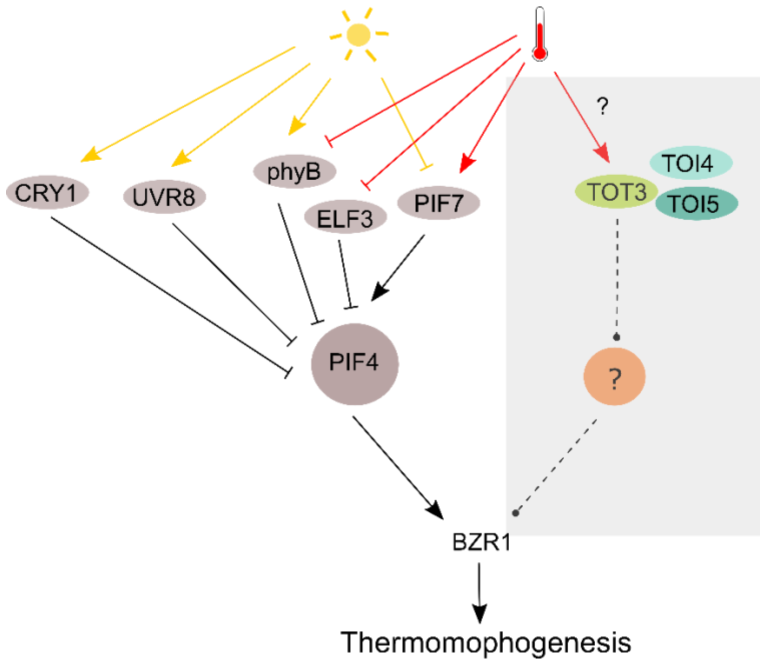

Supplementary Figure 25. Potential molecular model for light-independent and warm temperature-mediated pathway controlled by TOT3-TOI4-TOI5 and requiring BZR1 (grey). In the light-dependent pathway, hypocotyl growth during thermomorphogenesis is mainly controlled by PIF4, which is in turn regulated by or works in tandem with different thermosensors (phyB, ELF3, PIF7) and other light receptors (CRY1, UVR8). High temperature-activated PIF4 and auxin signalling will then activate the BR signalling pathway via BZR1 to promote thermomorphogenesis. In a parallel, PIF4-independent pathway, TOT3 – together with other MAP4Ks – perceives temperature information to regulate BR signalling by controlling BZR1 activity via an unknown intermediate regulator.

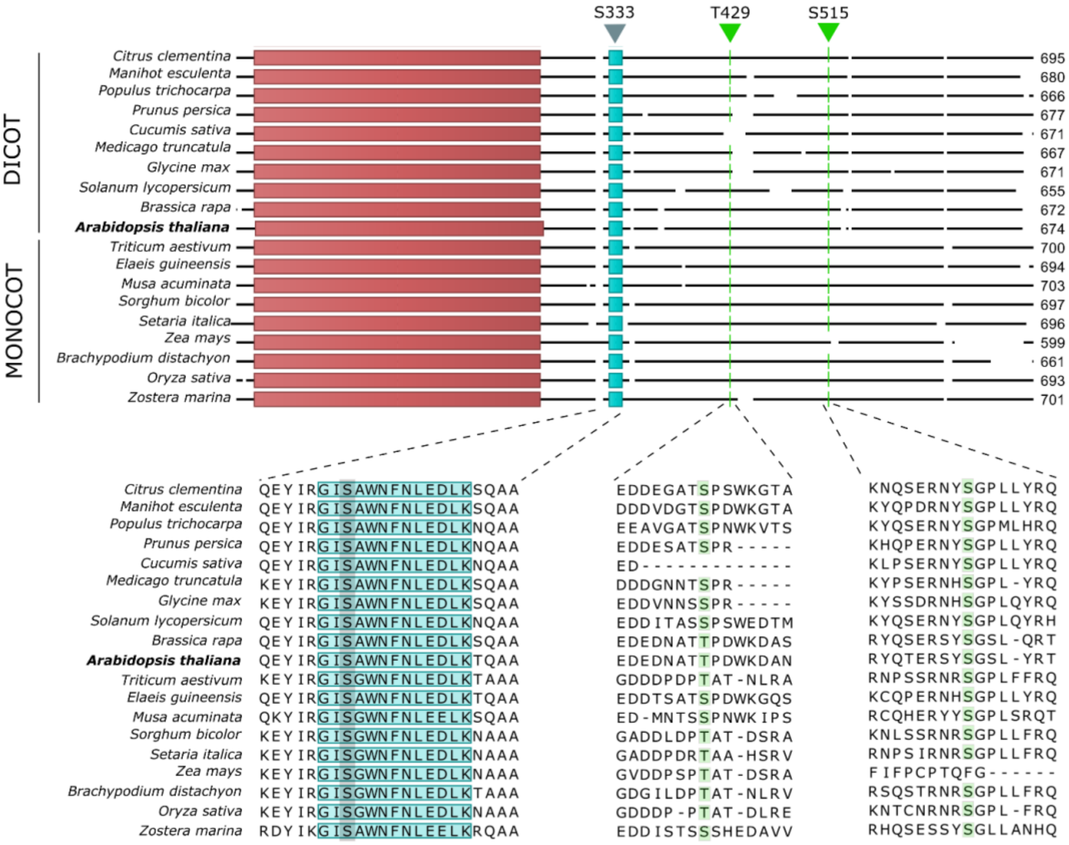

Supplementary Figure 26. Alignment of TOT3 orthologs in monocots and dicots. The kinase domains are marked in red. S333 of AtTOT3 and the corresponding phosphopeptides are highly conserved (blue) in both monocots and dicots. Two other phosphosites (and neighbouring amino acids) of AtTOT3 (green) reproducibly identified in phosphoproteome analysis are less conserved in the same set of species. TOT3 orthologs were identified using blastp in PLAZA 3.0. Sequence alignments were performed using CLC Workbench (v. 7.6.1). The following proteins were included in the figure: Ciclev10014466m.g, Manes.15G165300, Potri.001G349300, PPE_003G26360, Cucsa.011510, Medtr6g006770, Glyma19g01000.3, Solyc02g086790.2, Brara.J02001, AT5G14720, TraesCS7B02G130700, EGU1474G0610, MAC09G1534, Sobic.010G139600, Seita.4G148100, Zm00001d045967, Bradi1g42257, Os06g29120

Supplementary Tables

Supplementary Table 1. List of primers, nucleotide oligomers or short sequences used in this study.

Primer_ID	Sequence	Application	Note
ARP7-Fwd	ACTCTTCCTGATGGACAGGTG	RT-qPCR	Housekeeping gene
ARP7-Rev	CTCAACGATTCCATGCTCCT	RT-qPCR	Housekeeping gene
EF1α-Fwd	CTGGAGGTTTTGAGGCTGGTAT	RT-qPCR	Housekeeping gene
EF1α -Rev	CCAAGGGTGAAAGCAAGAAGA	RT-qPCR	Housekeeping gene
PIF4-Fwd	CCAGATCATCTCCGACCGGTTTG	RT-qPCR	
PIF4-Rev	CTAGTGGTCCAAACGAGAACCGT	RT-qPCR	
HSP70-Fwd	GAAGTACAAGGCTGAGGATGAAGAAC	RT-qPCR	
HSP70-Rev	CTTCTCGTCCTTGATCGTGTTCC	RT-qPCR	
IAA29-Fwd	TCCTCTGGAATCCGAGTCTTC	RT-qPCR	
IAA29-Rev	GGTGGCCATCCAACAACTT	RT-qPCR	
ATHB2-Fwd	TGCGAGTTCTTACGGAGATG	RT-qPCR	
ATHB2-Rev	AGTAGTGGGTGGGCTCATGT	RT-qPCR	
CPD-Fwd	TTGCTCAACTCAAGGAAGAG	RT-qPCR	
CPD-Rev	TGATGTTAGCCACTCGTAGC	RT-qPCR	
YUC8-Fwd	GACTGCTCGGTTCGATGAGA	RT-qPCR	
YUC8-Rev	TGAATCACCTCACCGGAAAA	RT-qPCR	
TOT3_before_Fwd	GAGATGGTGTCAGTGCGACT	RT-qPCR	
TOT3_before_Rev	ACCTCTCTCCGGATCCCATC	RT-qPCR	
TOT3_middle_Fwd	TGGTCATGCCCCATTTTCCA	RT-qPCR	
TOT3_middle_Rev	CCAGGCATGTACCCACCATT	RT-qPCR	
TOT3_after_Fwd	CAAGGGACGGTTTAAGGTCA	RT-qPCR	
TOT3_after_Rev	GATTGAGGCGGTTGTAGCAT	RT-qPCR	
TOI4-Fwd	CAAAATTCAATGCAGCAGGA	RT-qPCR	
TOI4-Rev	GTCAGATCCTCGAGGCAAAG	RT-qPCR	
TOI5-Fwd	ACCCATCATGCCTCAACTTC	RT-qPCR	
TOI5-Rev	AGCCACAGGTTCAACGTTTC	RT-qPCR	
TOT3_CDS_Fw	ATGGAATCGGGTTCAGAGAAAAAGT	PCR	to pick up *TOT3* CDS
TOT3_CDS_Rv	TCAATCATTTCTGTGTGTTAATGCGTT	PCR	to pick up *TOT3* CDS
TOT3_CDS_goldengate_Fwd	TTTTGGTCTCAGGCTCCATGGAATCGGGTTCAG	PCR	containing BsaI restriction site
TOT3_CDS_goldengate_Rev	TTTTGGTCTCACTGAATCATTTCTGTGTGTTAATGCGTTC	PCR	containing BsaI restriction site
TOT3_promoter_goldengate_Fwd	TTTTGGTCTCAACCTgaacaatatccagagcttg	PCR	containing BsaI restriction site
TOT3_promoter_goldengate_Rev	TTTTGGTCTCATGTTAATTTTTACTTTATAATCTTCTTATAGCATATATGC	PCR	containing BsaI restriction site

TOT3_D157N_F	gctgatactccaaagtttgctaacttaacggcaccat	PCR	site-directed mutangenesis for S333E
TOT3_D157N_R	atggtgccgttaagttagcaaactttggagtatcagc	PCR	site-directed mutangenesis for S333E
TOI4_GW_Fwd	AAAAAGCAGGCTCCACCATGGTGTCTCGGTTTCGTCTTGC	PCR	for gateway cloning into pDONR221
TOI4_GW_Rev	AGAAAGCTGGGTCTTACAATTGCTCGCGACCGGTG	PCR	for gateway cloning into pDONR221
TOI5_GW_Fwd	AAAAAGCAGGCTCCACCATGGTGGGAGGAGGAGGAGGTA	PCR	for gateway cloning into pDONR221
TOI5_GW_Rev	AGAAAGCTGGGTCTTAGTGTTCGCGACCCGTGA	PCR	for gateway cloning into pDONR221
tot3-1_F	TTGCATTATCCTCGTCCTCAC	PCR	genotyping for SALK_065417
tot3-1_R	CAAAAGATTCTCGAAAGTGCG	PCR	genotyping for SALK_065417
tot3-2_F	TAACATGGTGGGGAAGTGATC	PCR	genotyping for SALK_086087
tot3-2_R	AAAAGTGCAACAACGATCTGG	PCR	genotyping for SALK_086087
LB_salk	ATTTTGCCGATTTCGGAAC	PCR	generic left border primer for genotyping of SALK lines
TOT3_pDON_Fwd	AAAAAGCAGGCTCCACCATGGAATCGGGTTCAGAGAAAAAGTTC	PCR	TOT3-CDS with attB sites as product
TOT3_pDON_Rev	AGAAAGCTGGGTCTCAATCATTTCTGTGTGTTAATG	PCR	TOT3-CDS with attB sites as product
TOT3-296-trunc_Rev	AGAAAGCTGGGTCTCATGGAGGAAGACCATTTAGAATTG	PCR	For Gateway cloning of the N-terminal fragment (aa 1-296) of TOT3 CDS
TOT3-340-trunc_Rev	AGAAAGCTGGGTCTCACTCGAGATTGAAATTCCAAGC	PCR	For Gateway cloning of the N-terminal fragment (aa 1-340) of TOT3 CDS
TOT3_297_Fwd_gate	AAAAAGCAGGCTCCACCATGTTAGGTGATCGCTATAGACAA	PCR	For Gateway cloning of the N-terminal fragment (aa 297-674) of TOT3 CDS
TOI4-g1-F	ATTGCCATAAGTTTATAATCTTTA	CRISPR-Cas9	assembled into pGG-A-AtU6-B
TOI4-g1-R	AAACTAAAGATTATAAACTTATGG	CRISPR-Cas9	assembled into pGG-A-AtU6-B
TOI4-g2-F	ATTGCGATCGCTGCAATAGTAATC	CRISPR-Cas9	assembled into pGG-B-AtU6-C
TOI4-g2-R	AAACGATTACTATTGCAGCGATCG	CRISPR-Cas9	assembled into pGG-B-AtU6-C
TOI5-g1-F	ATTGCTCTGTCGACCATAGTCTTT	CRISPR-Cas9	assembled into pGG-C-AtU6-D
TOI5-g1-R	AAACAAAGACTATGGTCGACAGAG	CRISPR-Cas9	assembled into pGG-C-AtU6-D
TOI5-g2-F	ATTGCTAATGGAAGAAATAGGCCA	CRISPR-Cas9	assembled into pGG-D-AtU6-E
TOI5-g2-R	AAACTGGCCTATTTCTTCCATTAG	CRISPR-Cas9	assembled into pGG-D-AtU6-E
Cadenza1716 (W122*)_WT	cGagagctttgagaacttccC	alignment	checking the presence of the point mutation
Cadenza1716 (W122*)_Alt	cGagagctttgagaacttccT	alignment	checking the presence of the point mutation
Cadenza0256 (Q191*)_WT	gtaatcatagccatgcagttgttG	alignment	checking the presence of the point mutation
Cadenza0256 (Q191*)_Alt	gtaatcatagccatgcagttgttA	alignment	checking the presence of the point mutation
Cadenza1716 (W122*)_FW	TCTAATCAGTTTGAGCACAGAATAATGCAT	PCR	genotyping
Cadenza1716 (W122*)_REV	CCTTAATAGCATTAAAACTGTAGTGCCCTAAT	PCR	genotyping
Cadenza0256 (Q191*)_FW	ACAGTATGATGAGAGGAATCAGAAGAATGATT	PCR	genotyping
Cadenza0256 (Q191*)_REV	AAGGAGGCAAACCTAGAGTCAACT	PCR	genotyping

Supplementary Table 2. List of Golden Gate entry plasmids and destination plasmids for constructs generated in this study.

Construct	pTOT3::GFP:TOT3	pTOT3::GFP:TOT3^{D157N}	pTOT3::HA:TOT3 (309 - 674)	pFASTRK24GW *toi4toi5*
**Destination vector**	pPGA	pPGA	pPGA	pFASTRK24GW
**Entry vectors**	pGGA000 (pTOT3)	pGGA000 (pTOT3)	pGGA000 (pTOT3)	pGG-A-AtU6-B (TOI4 gRNA1)
	pGGB-GFP-C	pGGB-GFP-C	pGGB-3xHA-C	pGG-B-AtU6-C (TOI4 gRNA2)
	pGGC000 (TOT3-CDS)	pGGC000 (TOT3-CDS)	pGGC000 (TOT3$^{309-674}$)	pGG-C-AtU6-D (TOI5-gRNA1)
	pGGD002	pGGD002	pGGD002	pGG-D-AtU6-E (TOI5-gRNA2)
	pGG-E-G7T-F	pGG-E-G7T-F	pGG-E-G7T-F	pGG-E-G7T-F
	pGGF008	pGGF008	pGGF008	pGGF008

SUPPLEMENTARY REFERENCES

1 Letunic, I. & Bork, P. Interactive Tree Of Life (iTOL) v4: recent updates and new developments. *Nucleic Acids Res*. **47**, W256-W259, doi:10.1093/nar/gkz239 (2019).

2 Vu, L. D. et al. Temperature-induced changes in the wheat phosphoproteome reveal temperature-regulated interconversion of phosphoforms. *J. Exp. Bot.* **69**, 4609-4624, doi:10.1093/jxb/ery204 (2018).
