# Supplementary material for: The membrane-localized protein kinase MAP4K4/TOT3 regulates thermomorphogenesis
Source: Nat Commun. 2021 May 14;12:2842. doi: 10.1038/s41467-021-23112-0 (PMC8121802; doi:10.1038/s41467-021-23112-0)
Supplement: Supplementary file 3 — Descriptions of Additional Supplementary Files [file 41467_2021_23112_MOESM3_ESM.pdf]

Descriptions of Additional Supplementary Files

Supplementary Data 1
Description: List of identified phosphosites and significantly deregulated phosphosites from phosphoprofiling of Arabidopsis seedlings transferred to 27 °C within 1 h.

Supplementary Data 2
Description: List of identified phosphosites and significantly deregulated phosphosites from phosphoprofiling of soybean transferred to 35°C for 1 h.

Supplementary Data 3
Description: Lists of non-redundant AGI Locus Identifiers for differentially phosphorylated (unique and significant) Arabidopsis proteins and (available) Arabidopsis orthologs for differentially phosphorylated soybean or wheat proteins

Supplementary Data 4
Description: List of deregulated phosphosites in wheat spikelets and wheat leaves at 34 °C - data extracted from Vu et al. (2018) J Exp Bot 69(19):4609- 4624

Supplementary Data 5
Description: List of TOT3 interactors identified from TAP analysis and GFP immunoprecipitation and their corresponding enriched GO terms.

Supplementary Data 6
Description: List of identified phosphosites and significantly deregulated phosphosites from phosphoprofiling of tot3-2 seedlings at 28°C.

Supplementary Data 7
Description: List of identified TOT3, TOI4 and TOI5 phosphosites from transient expression in tobacco leaves

Supplementary Data 8
Description: List of phosphosites identified from in vitro kinase assay of TOT3 and kinase dead TOI4/TOI5
